# On-Chip Strained Germanium Lasers: A Review

**DOI:** 10.3390/nano16060356

**Published:** 2026-03-14

**Authors:** Ronghuan Liu, Weiqi Song, Zi-Wei Zheng

**Affiliations:** 1 Digital Industry Research Institute, Zhejiang Wanli University, Ningbo 315100, China; 2 School of Materials Science and Engineering, Wuhan Textile University, Wuhan 430200, China

**Keywords:** germanium nanowire, straintronics, photonic-integrated circuits, on-chip laser

## Abstract

The 100 GHz-class ultrafast photonic integrated circuit (PIC) positions itself as a promising technology in the post-Moore era, when the bandwidth limit of metallic interconnections constrains current electronic integrated circuits. Nevertheless, the lack of an effective on-chip, CMOS-compatible laser source challenges the ongoing development of PIC. Germanium straintronics facilitate bandgap transformation from indirect to direct, thereby enabling effective band-to-band radiative recombination. Some parameters, such as nanowire diameters or crystalline orientation and strain direction, have a profound effect on the bandgap transformation of Ge nanowires. In this review, we will discuss changes in the fundamental physical properties of Ge nanowires under strain, including mechanical, electronic, optical, and thermal properties. Subsequently, we summarize common methods for strain engineering, as well as novel approaches that have emerged in recent years. Some notable application cases reported in the last few decades will be discussed in detail. This review may fill knowledge gaps and provide a solid background for forthcoming investigations of on-chip strained Ge lasers.

## 1. Introduction

Nowadays, the leading-edge transistor channel length has been shrunk to the physical size limit (i.e., the state-of-the-art 2 nm technology node), underscoring the enormous success of the electronics industry and the arrival of the post-Moore era. The node naming “2 nm” normally referred to the technology generation rather than the physical gate length. The 2 nm node normally utilizes the 3D Gate-All-Around FET architecture with a physical gate length of approximately 12–15 nm, in order to mitigate tunneling effects. Nevertheless, the shorter transistor channel length also results in significantly higher transistor and integration densities, thereby prompting longer interconnect metal wires with smaller diameters (e.g., sub-10 nm). Such tiny metal wires inevitably exhibit a sharp increase in resistance, accompanied by a serious bandwidth limitation due to the RC time decay. The loss at ultrahigh frequencies is unacceptably large in the compact metal wires as well. Furthermore, signal cross-talk between adjacent interconnect metallic wires occurs due to wave reflections on the lines. Additionally, other problems, including undesired heat generation and reliability concerns, challenge advanced CMOS fabrication technologies that follow Moore’s law. Therefore, in the post-Moore era, the power-performance-area-and-cost scaling strategy aims to find a balance point among the trade-offs among four parameters. However, the inherent physical limits of electronic integrated circuits make it difficult to realize a 100 GHz-class ultrafast computing chip. Compared to electrical signals, optical signals typically operate at higher carrier frequencies, thereby achieving impressively higher data-carrying capacity and greater bandwidth. Please note that “100 GHz” refers to the intrinsic response speed or target optical bandwidths required for next-generation interconnects, instead of the clock frequency of digital logic. Furthermore, the lower resistive losses for light propagation in optical waveguides or fibers facilitate more efficient signal communication with lower energy consumption. From this perspective, photonic integrated circuits should be among the next-generation technologies after electronic integrated circuits in the post-Moore era.

In principle, a photonic integrated circuit system commonly comprises several essential components: on-chip lasers, waveguides, modulators, and photodetectors, which correspond to the roles of generation, propagation, modulation, and detection of optical signals, respectively. Aside from on-chip laser sources, the other three components based on Si materials have been extensively studied and reported [1,2] because they do not require a direct bandgap to enable band-to-band radiative recombination. Si’s indirect bandgap makes it difficult to manufacture on-chip laser sources, posing a major impediment to the practical advancement of photonic integrated circuits. Currently, on-chip lasers commonly utilize the Group III–V; direct-bandgap semiconductors via heterogeneous integration. Poor compatibility with complementary metal-oxide semiconductor (CMOS) technology blocks the integration of Group III–V; semiconductors onto the Si substrate [3,4]. For example, lattice mismatch causes defects and dislocations at the interface between the Group III–V; semiconductor epitaxial layer and the Si substrate. Cross-contamination is another issue that poses a risk of polluting standard Si foundry tools, as Group III–V; elements often serve as deep-level traps in Si and thereby degrade Si transistor performance. In contrast, germanium (Ge) is a Group IV element with inherent CMOS compatibility, allowing the monolithic integration with existing Si-based CMOS infrastructure. Therefore, this creates a strong motivation to develop monolithic CMOS-compatible Ge on-chip laser sources.

**Scheme 1:**
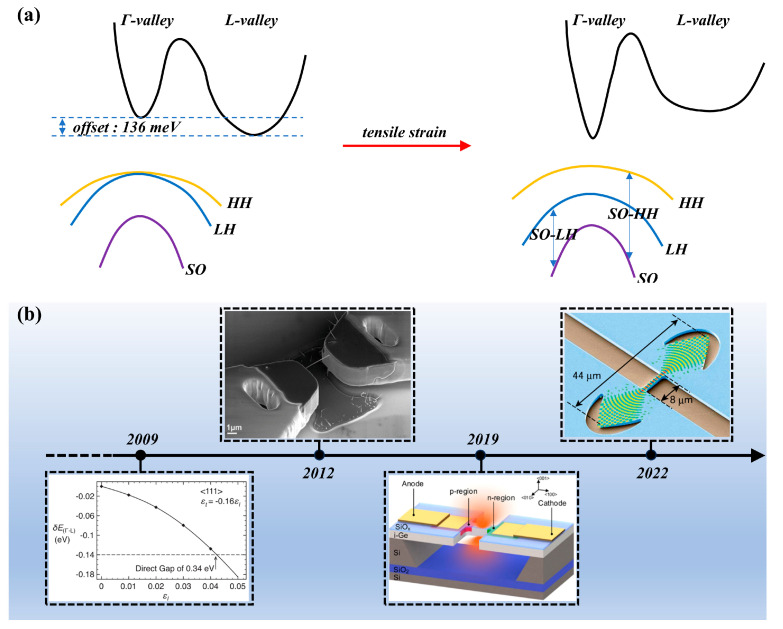
(**a**) The left panel presents the energy band structure of strain-free Ge and the right panel shows that of the strained Ge. HH, LH, and SO denote the heavy-hole (the yellow line), light-hole (the blue line), and split-off bands (the purple line), respectively. The double-headed arrows indicate the intervalence-band transition for SO-LH and SO-HH. (**b**) Historical milestones in strained Ge laser research. Four panels (from left to right) are adapted from [4,5,9,10].

In Group IV, Si exhibits an indirect bandgap of E_g_ = 1.2 eV and a much higher direct bandgap of E_ᴦ1_ = 3.4 eV at 300 K. In contrast, Ge demonstrates a small difference of only 136 meV between the indirect and direct bandgap (i.e., E_g_ = 0.66 eV and E_ᴦ1_ = 0.8 eV at 300 K). From this perspective, the conversion from the indirect to direct bandgap in Ge via strain engineering is promising for realizing CMOS-compatible laser sources for photonic integrated circuits (see Scheme 1). In 1955, R. G. Treuting was the first to report the strain engineering for bulk Ge [3]. Due to the increasing demand for on-chip lasers in photonic integrated circuits, the strain engineering of Ge nanowires has been intensively investigated over the last two decades. In 2009, Feng Zhang et al. theoretically predicted that uniaxial tensile strain could induce indirect-to-direct bandgap conversion in Ge nanowires [4]. In 2012, J. Greil et al. systematically studied the fundamental electro-optical properties of strained Ge nanowires [5]. In 2013, Donguk Nam et al. observed a large enhancement of photoluminescence of >200 times under 2.3% tensile strain [6]. In 2017, Shuyu Bao et al. achieved a 1.6% tensile strain and a 1530 nm near-infrared laser at 83 K, with a low optical pumping threshold density of ~3.0 kW [7]. In 2019, F.T. Armand Pilon et al. demonstrated a mid-infrared Ge nanowire laser under ultrahigh-tensile strain of 5.9%, with an excellent internal quantum efficiency of approximately 100% at 20 K [8]. Aside from optically pumping, Jialin Jiang et al. demonstrated an electrically driven Ge nanowire laser with a broad near-infrared emission peak (from 1580 nm to 1885 nm) at room temperature [9]. In 2022, FT Armand Pilon et al. advanced beyond strain engineering by combining it with n-type doping [10]. (See historical progress in Scheme 1.)

These works demonstrate that tensile strain is an encouraging strategy for Group IV lasers. Some reviews have been published in this area, and the effect and importance of tensile strain in Ge lasers have often been highlighted [11]. To the best of our knowledge, the literature may currently lack a review that systematically summarizes the underlying physical properties of strained Ge, specifically under the ultrahigh-tensile strain regime. This article distinguishes itself by providing an up-to-date overview of the high-tensile strain regime, in which the physical properties of Ge exhibit significant nonlinearities in bandgap narrowing and Raman shifts. We will discuss the fundamental properties under ultrahigh strain to provide an overview of the effects of strain. Compared with previous reviews, we summarize and discuss the fabrication methods in terms of both physical mechanisms and their scalability for mass production. Bridging the gap between fundamental physics and practical device application positions this work as a clear roadmap for the achievement of CMOS-compatible monolithic strained Ge lasers.

## 2. Fundamental Physical Properties

### 2.1. Mechanical Properties

**Figure 1:**
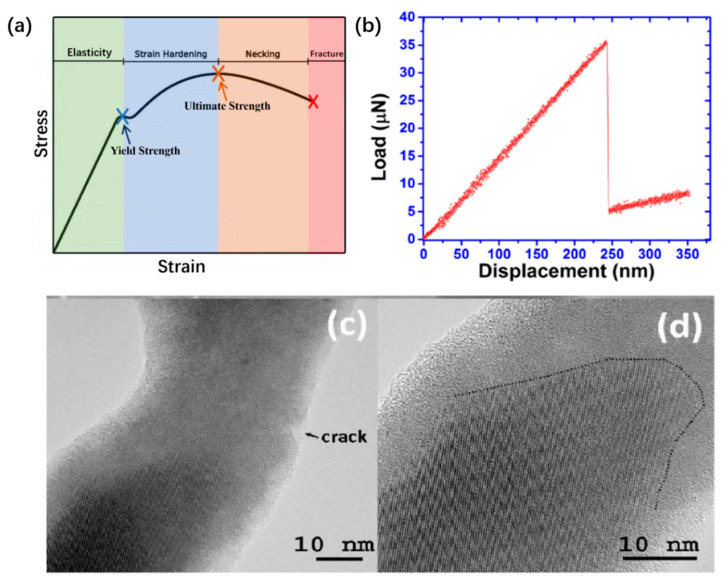
(**a**) A typical stress–strain curve of tensile nanowires. Elasticity (the green region), strain hardening (the blue region), necking (the orange region) and fracture (the red region) are the four main regions. The cross marks highlight the boundary between two adjacent regions. In the elasticity region, the slope represents the Young’s modulus. (**b**) A load–displacement curve of Si nanowires. The load represents the stress applied to Si nanowires, and the displacement is the deformation of one terminal of a Si nanowire while another terminal is unchanged. The abrupt drop before the fracture indicates the brittle properties of Si. Adapted with permission from Ref. [19], Copyright © 2016, The Authors. CC-BY-4.0 (https://creativecommons.org/licenses/by/4.0/). (**c**) High-resolution transmission electron microscopy (HRTEM) of ultrahigh strained Ge nanowire. A crack exists clearly near the edge of the Ge nanowire. (**d**) Corresponding HRTEM image of the crystalline area, but the strain-induced amorphous phase surrounds this area. Both panels (**c**,**d**) were adapted with permission from Ref. [20], Copyright © 2010, American Chemical Society.

For bulk Ge, a tensile strain of 1% can easily cause fracture, corresponding to a fracture strength of 40~95 MPa. Unlike the bulk counterparts, Ge nanowires can tolerate a highly tensile strain of ~17% and the ideal fracture strength of 14~20 GPa [12,13] due to the size effect and self-purification of nanowires [14,15,16]. From this perspective, Ge nanowires are a promising platform for achieving a direct-bandgap Ge laser under high strain. Normally, the mechanical performance of solid nanowires should follow the stress–strain law, including elasticity, strain hardening, necking, and fracture, as shown in Figure 1a. At room temperature, bulk Ge is brittle, exhibiting only elastic behavior before fracture [17]. Nevertheless, at high temperatures, bulk Ge exhibits plastic deformation before fracture, which is non-recoverable and permanent deformation [18]. Figure 1b reveals that the nanowire can undergo elastic deformation without a visible sign of plastic deformation before fracture [19]. The high elastic limit of Si NWs originates from the good crystalline and atomically smooth surfaces. Nevertheless, plastic deformation at room temperature may occur if the nanowire’s surface quality is insufficient. For example, Brain A. Korgel et al. found plasticity in 〈111〉-oriented Ge nanowires under ultrahigh strain [20]. In Figure 1c,d, a crack happens in the amorphous region before fracture, suggesting plastic deformation.

Young’s modulus and the Poisson ratio are two key parameters used to characterize elastic properties. In the elasticity region, strain is proportional to stress, and Young’s modulus E is given by

(1) E=σε

where σ is the uniaxial stress, defined as the applied force divided by the cross-sectional area, with a unit of N/m^2^. ε is the strain, following

(2) ε=δL0

with δ the geometry deformation and L_0_ the initial length of the nanowire without strain. On the microscopic scale, Young’s modulus presents the strength of interatomic forces between adjacent atoms. Therefore, Young’s modulus should be relative to crystal orientation. For example, the Young’s modulus of bulk Si in the crystal orientation of (100), (110) and (111) is 202 GPa, 169 GPa and 187 GPa, respectively [21]. Aside from the impact of crystal orientation, the Young’s modulus of nanowires is size-dependent as well, due to the enhanced surface-to-volume ratio [22]. Yong Zhu et al. reported that the Young’s modulus of (111)-oriented Si nanowires decreases from 187 GPa to 90 GPa, while the diameter of nanowires decreases from 30 nm to 10 nm [23]. The possible theories elucidating the size-dependent Young’s modulus include (i) the impact of the surface oxide layer [24,25]; (ii) nonlinear elastic response of the nanowire core [26]; and (iii) surface stress and surface elasticity [27,28].

Another critical parameter is the Poisson ratio, which usually describes the geometric deformation of tensile nanowires:

(3) $$\nu =-\frac{\varepsilon_{⊥}}{\varepsilon_{∥}}$$

where ε_﬩_ is the traverse strain perpendicular to nanowires and ε_ǁ_ is the longitudinal strain along the axis. The sign “−” indicates that the cross-sectional area would compact while the length expands under the tensile strain along the axis. Similar to Young’s modulus, the Poisson ratio of nanowires is relevant to crystal orientation and nanowire diameter as well. Song Li et al. reported that compression strain-induced lattice variation distribution is isotropic for 〈100〉- and 〈111〉-oriented nanowires, but anisotropic for 〈110〉-directed ones [29]. Additionally, Xiao Zhang et al. demonstrated that a 〈110〉-oriented Ge nanowire with a smaller diameter should have a smaller Poisson ratio [30]. The size-dependent Poisson ratio of nanowires can be well explained via the core–shell model with an elastically stiffer surface layer and a bulk-like core [31,32].

### 2.2. Electronic Properties

**Figure 2:**
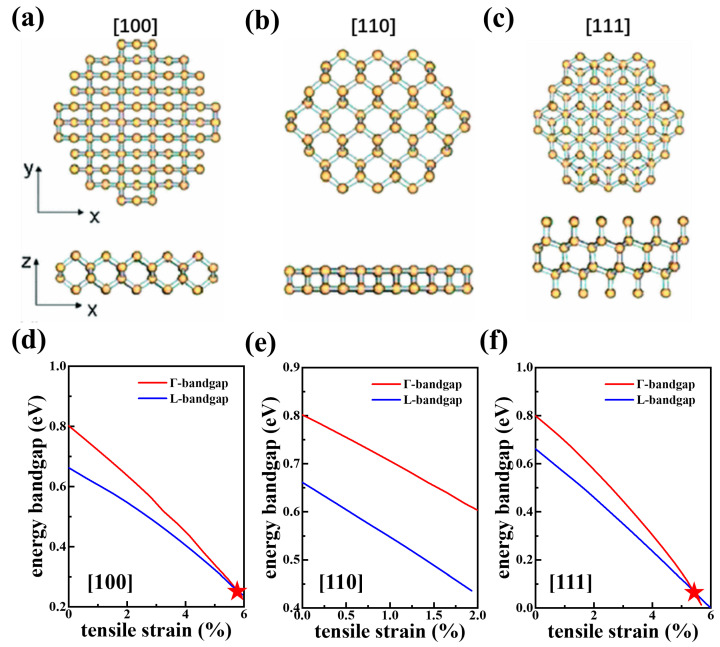
(**a**–**c**) The atomic structure of typical diamond-structure nanowires along three main crystallographic directions, namely <100>, <110> and <111>. The upper panels are the cross-section view and the lower panels are the side view. Adapted with permission from Ref. [34]. Copyright © 2008, American Chemical Society. (**d**–**f**) The bandgap induced by different uniaxial strain and crystal orientations. The red star indicates the minimum strain required to realize the direct bandgap. Data extracted and replotted from Ref. [35].

Tensile strain can alter the lattice profile, resulting in a different band structure in semiconductors and changes in electronic properties. Figure 2a–c illustrate the atomic structures of Ge in the various crystal orientations [33]. The periodic unit cells of Ge nanowires along three orientations depict different profiles in the cross-section view (i.e., square, hexagonal and parallelogram shapes for 〈100〉, 〈110〉 and 〈111〉 orientations, respectively).

Some simulated methods have been developed to compute band structure, such as deformation potential theory (DPT), Keating’s model (KM), and tight-binding formalism (TBF) [35,36,37,38,39,40,41,42]. Deformation potential theory is the most common technique, and many related results have been reported [43,44]. Nevertheless, deformation potential theory may not be accurate for tensile strains exceeding 1% [35]. DPT often assumes a linear relationship, but the actual electronic band structure of strained Ge exhibits a nonlinear dependence under high strain. Jose M. Escalante reported that the results from KM + TBF were more accurate than DPT at high tensile strain [35]. Additionally, the Γ-bandgap decreases linearly at low strain, but becomes nonlinear under high tensile strain (see Figure 2d–f). A degeneration of the L-valley is not split when the tension is applied on Ge nanowires along 〈111〉, whereas the L-valley of Ge nanowires along 〈100〉 and 〈110〉 splits into four sub-bands under tensile strain.

Table 1 demonstrates the predicted threshold values required to convert the indirect band of Ge into the direct gap. These theoretical results indicate that 〈111〉-oriented Ge nanowires are the most efficient and promising configuration for achieving a direct gap. The orientation 〈110〉 was reported to be unable to induce a direct gap.

**Figure 3:**
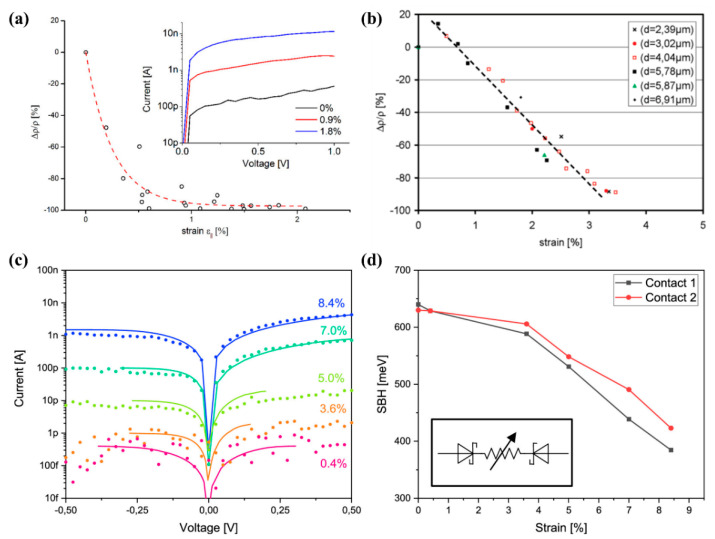
(**a**) Relative changes in resistivity versus the applied strain of <111>-oriented Ge nanowires. The inset shows current–voltage curves under different strains. The dots and dashed line represent the experimental data and the corresponding fitting curve, respectively. Adapted with permission from Ref. [5]. Copyright © 2012, American Chemical Society. (**b**) Relative changes in resistivity under various strains. All the diameters of the <111>-oriented Si nanowires are about 100 nm but their original length d is different, as shown in the legend. Adapted with permission from Ref. [50]. Copyright © 2010, American Chemical Society. (**c**) I/V curves (dots) of the Au-Si-Au back-to-back Schottky diode under various strain conditions. Solid lines demonstrate fitting curves based on the formula $V=V_{1}+V_{2}+R_{NW}AJ=\frac{n_{1}k_{B}T}{q}\ln\left(\frac{J}{J_{0}}+1\right)+\frac{n_{2}k_{B}T}{q}\ln\left(\frac{J}{J_{0}}+1\right)+R_{NW}AJ$, with k_B_ the Boltzmann constant, T the temperature, n the ideality factor, q the element charge, A the contact area to Au electrodes and R_NW_ the resistance of Si nanowires. (**d**) Effective Schottky barrier height (SBH) of the two back-to-back Au-Si contacts as the function of strain. The inset depicts the circuit model of the back-to-back Schottky diode. Panels (**c**,**d**) are adapted with permission from [5,51]. Copyright © 2024, American Chemical Society.

Tensile strain can modulate the electrical resistivity of Ge nanowires as well. Under the applied strain ε_L_, the relative resistance change ΔR/R_0_ follows

(4) $$\Delta \rho /\rho_{0}=∆R/R_{0}-\left(1+2v\right)\varepsilon_{L}$$

with ν the Poisson’s ratio. Ge nanowires without any doping normally exhibit p-type conductivity, due to the surface doping from the native germanium oxide [49]. The inset of Figure 3a shows the current enhancement via increasing the applied tensile strain. The corresponding relative changes in resistivity are summarized in Figure 3a. Under the tensile strain, the reduction in the bandgap can increase the carrier concentration, thereby decreasing the resistivity [50]. Additionally, under the highly tension-strained conditions, the Γ valley becomes lower than the L valley, enabling the direct bandgap and electron mass reduction, as the electrons in the L valley exhibit a heavier effective mass than those in the Γ valley. Thus, increased mobility may be another factor contributing to the decrease in resistivity. Similar phenomena also appear on the strained Si nanowires, as shown in Figure 3b [7,51].

Aside from nanowire resistivity, the Schottky barrier at the electrical contact also depends on the strain levels. Maximilian G. Bartmann et al. systemically investigated the impact of ultrahigh strain on the Au-Si Schottky barrier. Figure 3c demonstrates the current–voltage characteristics of the Au-Si-Au back-to-back Schottky diode under different strain conditions. The saturation current increases with strain magnitude due to the lowered Schottky barrier height (Figure 3d) and the narrowed bandgap.

### 2.3. Optical Properties

**Figure 4:**
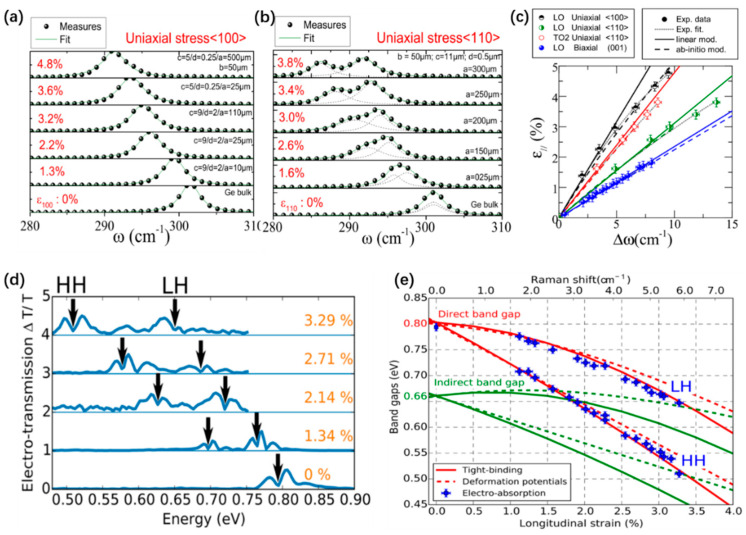
(**a**) The Raman spectra of Ge micro-bridges with <100> under various uniaxial stress. (**b**) The Raman spectra of Ge micro-bridges with <110> under various uniaxial strains. (**c**) The experimental data are compared with the theory model. The dots are obtained from micro-XRD measurements, dashed lines are calculated by ab initio models, and straight lines represent the linear coefficients of Raman shift. Panels (**a**–**c**) adapted with permission from Ref. [52]. Rights managed by AIP Publishing. (**d**) Electro-absorption spectra of strained Ge micro-bridges measured under unpolarized light. (**e**) Bandgaps of strained Ge micro-bridges depend on longitudinal strain, which is calculated by wavelength shift in Raman spectroscopy. Blue squares represent electro-absorption measurements compared with theoretical values from deformation potentials (dashed red line) and the tight-binding model (solid lines). The upper curve is the bandgap from the light-hole band, and the lower one corresponds to the heavy-hole band. Panels (**d**,**e**) adapted with permission from Ref. [53]. Copyright © 2016, American Chemical Society.

The Raman spectrum is an effective method for measuring the magnitude of tensile deformation. If the applied tensile strain is not high, the shift of the Raman peak will change linearly with strain. Once the strain value is too large, the linear relation will be incorrect in Figure 4a–c. For example, A. Gassenq et al. found that for the maximum reported Raman spectral shift, the expected strain value according to the usual linear model should be 4.2%, while only 3.8% is measured [52]. To get the correct model, they use theory and experiment to confirm the following formalism:

(5) $$\varepsilon =a\times ∆\omega +b\times ∆\omega^{2}$$

where a and b are constants, and Δω is the value of the peak shift with the strain ε.

A similar phenomenon is also revealed through optical electro-absorption measurements that use the Franz–Keldysh effect to extract the band gap of semiconductors [53]. Under the tensile strain, the valence band will be split into the heavy-hole band (HH) and the light-hole band (LH), and the greater stress will enlarge the difference between HH and LH, which is clearly revealed in Figure 4d. A high strain forces the Ge band to transfer from indirect to direct, thereby creating direct transitions (Γ→HH/LH) for effective light absorption. As the direct transitions (Γ→HH/LH) are more efficient than phonon-assisted indirect transitions (L→HH/LH) on light absorption, the sharp onset in the absorption spectra should originate from direct transitions. In other words, the weaker indirect absorption tail could be regarded as a background signal. Thus, the corresponding energy in Figure 4e demonstrates direct transitions (Γ→HH/LH). Moreover, deformation potentials from the model-solid theory were compared with experimental data, but the agreement was broken beyond 2.0% [54]. The tight-binding model with a second-order amendment showed a good agreement with the experimental data, indicating the importance of the amendment for the highly strained Ge. In particular, owing to the significant response between the heavy-hole band and tensile uniaxial stress, the relation between longitudinal strain and the band gap can be expressed as

(6) $$E_{g\Gamma }\left(\varepsilon_{100}\right)\left(eV\right)=0.80-\left(7.31\pm 0.15\right)\varepsilon_{100}-\left(37\pm 7\right){\varepsilon_{100}}^{2}$$

where *E*_*g*Γ_ is the bandgap from the top of the heavy-hole band to the bottom of Γ-valley and *ε*_100_ is the tensile strain along 〈100〉.

### 2.4. Thermal Properties

**Figure 5:**
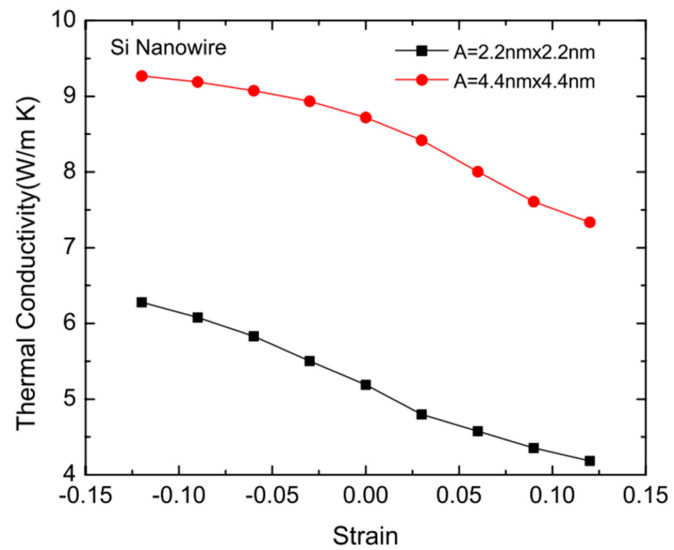
The thermal conductivity of Si nanowires as a function of strain. Adapted with permission from Ref. [55]. Copyright © 2010, American Physical Society.

Effective thermal management is critical to achieving a stable strained-Ge laser (discussed in further detail in Section 5.2). Ronggui Yang et al. reported that the thermal conductivity of Si nanowires drops from compressive to tensile strain in Figure 5 [55].

The thermal conductivity measures a material’s ability to transfer heat. The characteristic size of the phonon mean free path (MFP) for Ge and Si is in the range of 10–1000 nm at 300 K [56,57]. When the diameter of the nanowires is smaller than MFP, the thermal conductivity (k) would be suppressed owing to phonon boundary scattering and surface roughness [58,59,60,61,62]. In particular, at 300 K, the thermal conductivity of bulk Ge and Si is 60 and 150 W/(m∙K), while that of their nanowires with a diameter of 19 nm is just 2.26 and 22 W/(m∙K), respectively [63]. Strain can change the phonon vibrational frequencies of a semiconductor, further influencing its thermal properties [64,65], because the lattice thermal conductivity is determined by heat capacity, phonon scattering rates, and phonon group velocities, all of which would be different under the changed vibrational frequencies. Heat transport is well known to be strongly related to phonon interactions with boundaries. The rate of boundary scattering changes linearly with the quantity of the phonon velocity that is normal to the boundary [66,67]. Compared with bulk counterparts, the surface interactions of Ge nanowires, owing to their high volume-to-surface ratio, are more important, and the strain effect on thermal conductivity will be stronger.

## 3. Strain Methods for Ge Nanowires

In recent decades, scientists have applied various methods to achieve tensile-strained Ge or Si nanowires, such as mechanical manipulation [68], constructing micro- or nano-structures for geometric amplification [69], and coating with a silicon nitride stressor layer [70,71,72].

### 3.1. Mechanical Manipulation

**Figure 6:**
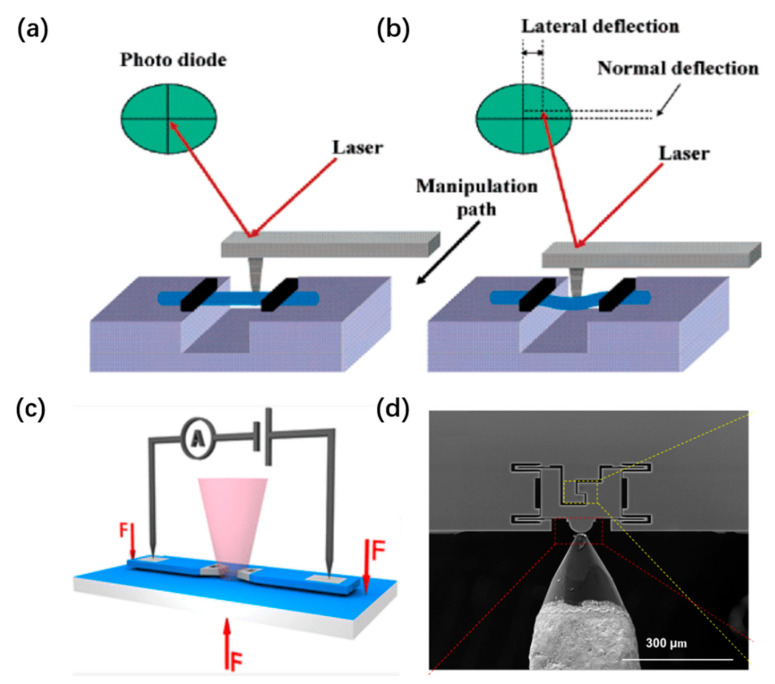
The strain force from the external probe. The process of the straining manipulation: (**a**) the original state and (**b**) the working state. The center of the Ge nanowire is suspended over the substrate, and a tip is used to apply strain. The lateral and normal deflection signals of light are recorded by photodiodes. Panels (**a**,**b**) adapted with permission from Ref. [68]. Copyright © 2006, American Chemical Society. (**c**) The structure of the 3-point straining module, where two downward forces on the two sides and an upward force at the center lead to the bend formation of suspended single nanowires. Adapted with permission from Ref. [5]. Copyright © 2012, American Chemical Society. (**d**) Scanning electron microscopy (SEM) of the push-to-pull micromechanical plate. A single nanowire is bonded between the gap shown in the yellow rectangle. The tip shown in the red rectangle is the tool applying the strain. As the tip moves toward the top, the Si nanowire will be under tensile stress. Adapted with permission from Ref. [19], Copyright © 2016, The Authors. CC-BY-4.0 (https://creativecommons.org/licenses/by/4.0/).

Mechanical manipulation is a very common method for applying tensile strain. In 2006, John J. Boland et al. reported a kind of atomic force microscope (AFM) manipulation [68]. Nanowires were clamped over trenches on a SiO_2_ substrate, and the resulting lateral load was measured from an AFM tip (Figure 6a,b). This approach can accurately detect well-defined clamping points and eliminate the effects of wire–substrate friction. In 2012, Alois Lugstein et al. bent a Ge nanowire by a sophisticated micromechanical 3-point strain module (Figure 6c) [5]. In 2016, Hongti Zhang et al. presented a push-to-pull micromechanical device (MMD) actuated by an external quantitative nano-indenter. The MMD is a free-standing configuration, including a semicircular end and specifically designed cutouts, as depicted in Figure 6d [19]. When a diamond punch pushes the semicircular end, the trench width between the two arms increases, thereby providing uniaxial tension to the nanowires deposited atop the trench. Although mechanical manipulation technologies readily achieve ultrahigh strains, integrating external mechanical operation tips with micro-structure cutouts remains a significant challenge.

### 3.2. Geometric Amplification

**Figure 7:**
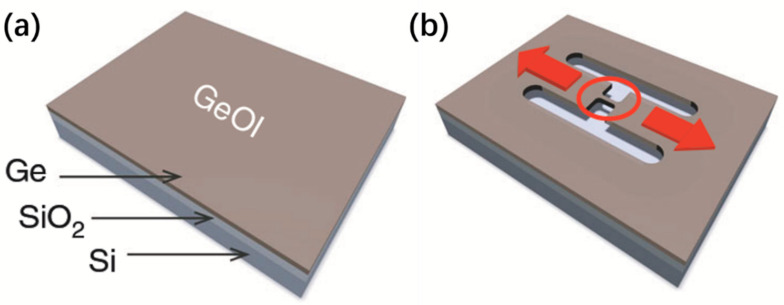
The strain force is induced by thermal expansion mismatch. (**a**) The structure of germanium-on-silicon (GOI) wafer. A small strain exists between the Ge and SiO_2_ layers. (**b**) The model of the common micro- or nano-structure. The suspended micro-bridge is shown in the red circle. The red arrows represent the direction of applied strain. Panels (**a**,**b**) adapted with permission from Ref. [8]. Copyright © 2019, Springer Nature. CC-BY-4.0 (https://creativecommons.org/licenses/by/4.0/).

Another popular approach is to build suspended micro- or nano-structures via common top-down techniques. At room temperature (300 K), the thermal coefficient of Si, SiO_2_ and Ge is $2.6\times 10^{-6}\;{{}^{\circ}C}^{-1},5\times 10^{-7}\;{{}^{\circ}C}^{-1}$ and $5.8\times 10^{-6}\;{{}^{\circ}C}^{-1}$, respectively. Due to the distinct differences in the thermal coefficients of Si, SiO_2_, and Ge, the relaxation of the Ge layer would be hindered during cooling, eventually causing a biaxially tensile-strained Ge layer. In other words, the strain exists between two different kinds of materials in nature. However, the strain is not high; it is typically 0.15% for both Ge/Si and Ge/SOI substrates [73], and the value is too small; focusing the dispersed strain on a point or line is an advisable approach to achieve high strain. In Figure 7, the micro- or nano-structure comprised pre-strained pads on both sides and the suspended micro-bridge in the center. The strain is achieved by relaxing two biaxially pre-strained pads and is applied intensively to the micro-bridge. The advantage of this method is its CMOS compatibility, but it requires two large pre-strained pads to achieve high strain (typically 100~300 µm in length) [74]. As far as we know, the size of the advanced MOSFET has now been reduced to several nanometers [75]. The large, strained laser seems to pose a challenge for high-density integration.

### 3.3. Stressor Layers

**Figure 8:**
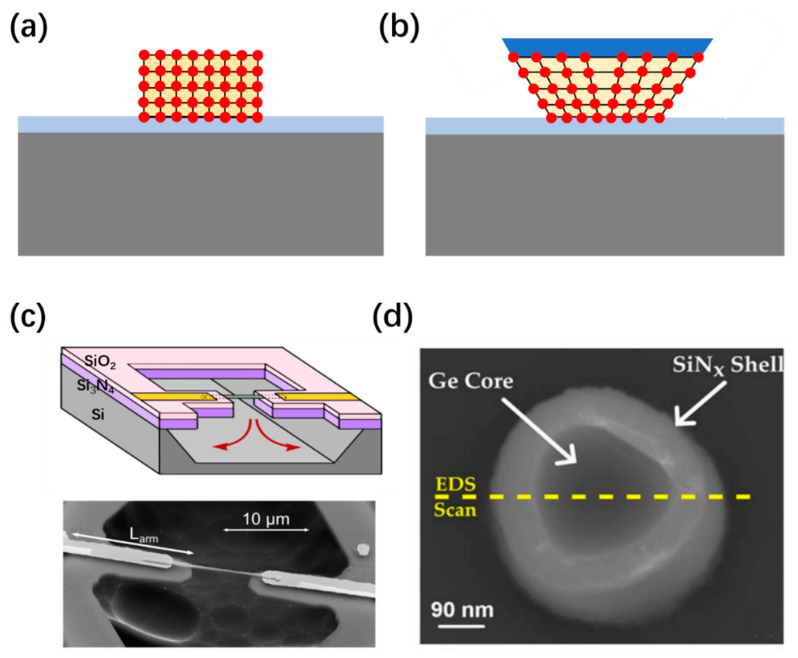
(**a**) The crystalline semiconductor nanowire on an SOI wafer. (**b**) A silicon nitride (SiN_x_) stressor layer is deposited on the top of the semiconductor nanowire to provide the tensile strain. (**c**) The suspended micro-structure with a Si_3_N_4_ stressor layer. The Si_3_N_4_ stressor layer is located between the Si substrate and the top SiO_2_ layer to provide a tensile strain. The upper image shows the schema of a strained structure using a Si_3_N_4_ stressor layer, and the lower image demonstrates the corresponding SEM image of a stressed nanowire. Adapted with permission from Ref. [77]. Copyright © 2015, American Chemical Society. (**d**) SEM image of the Ge-SiN_x_ core–shell nano-structure in the cross-sectional view. In this case, the SiN_x_ stressor layer is directly deposited on the sidewall of the Ge nanowires. Adapted with permission from Ref. [78]. Copyright © 2014, WILEY-VCH Verlag GmbH & Co. KGaA, Weinheim.

Using a silicon nitride (Si_3_N_4_) stressor layer is also a common strategy for obtaining tensile strain. Si_3_N_4_ is known to be a convenient stressor for other materials such as silicon. The amorphous Si_3_N_4_ layer is compressively strained, that is, it tries to expand the underlying structure in both horizontal directions, as shown in Figure 8 [76]. Vincent Calvo et al. grew a Si3N4 layer on the Si cavity to provide tensile strain [77]. The Si_3_N_4_ layer was etched into a rectangle and partially suspended from the Si substrate, as shown in the silicon nitride arms in Figure 8. The longer arm would generate higher strain, resulting in a larger deformation of the Ge nanowire at the center. For example, a longitudinal strain of 1.48 ± 0.01% was obtained with 14 µm arms. Denis Buttard et al. reported the core–shell Ge-SiN_x_ structure, where the Si_3_N_4_ was directly sputtered on the sidewall of the grown Ge nanowires [78]. The Ge nanowire diameter and the SiNx shell thickness were found to be effective parameters for controlling strain.

### 3.4. Other Fabrication Methods

**Figure 9:**
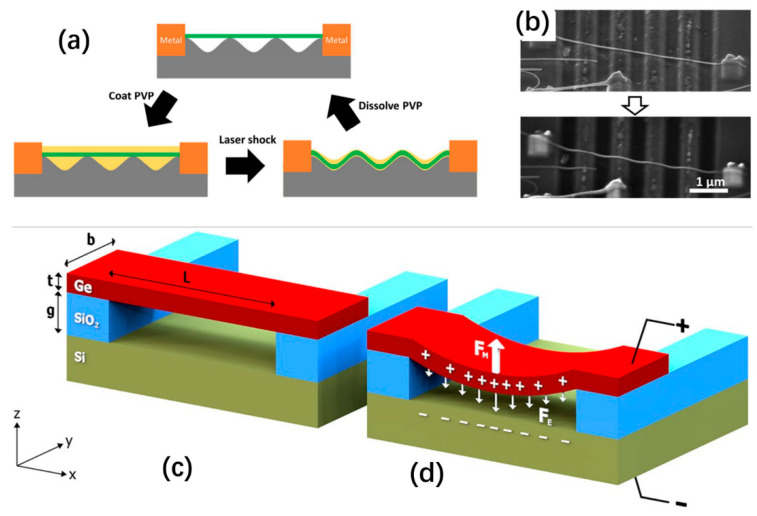
(**a**) The fabrication process of strained Ge nanowires by laser shock. A single Ge nanowire is deposited on the etched Si template with the empty nanogap arrays and then coated with PVP. Applying laser shock converts the Ge nanowire into a tensile-strained state, and dissolving PVP can return the strained Ge nanowire to its intrinsic state. (**b**) The corresponding SEM images of the first two steps of the fabrication process. The upper one shows the intrinsic Ge nanowire coat with PVP, and the lower one is the tensile-trained state after laser shock. Panels (**a**,**b**) adapted with permission from Ref. [79]. Copyright © 2016, American Chemical Society. (**c**) The original and (**d**) the strained Ge nanobeam in the suspended micro-structure. In this case, applying voltage is predicted to strain the Ge nanobeam. Panels (**c**,**d**) adapted with permission from Ref. [80]. Copyright © 2019, Springer Nature. CC-BY-4.0 (https://creativecommons.org/licenses/by/4.0/).

Some novel methods were also reported in recent years. Yaowu Hu et al. reported a laser shock elastic straining approach to achieve 3D tensile straining [79]. Laser shock is an ultrafast method for applying strain, but Ge nanowires are typically brittle and easily break when strains exceed their limit. To avoid nanowire fracture, polyvinylpyrrolidone (PVP) served as a cushion for nanowire shaping and an elastic strain preserver. As shown in Figure 9a, the Ge nanowire was transferred onto the Si wafer with the mold surface, then coated with PVP. Finally, a nanosecond laser was applied to induce deformation in the Ge nanowire. If the PVP coating film is removed, the Ge nanowire would become straight again. The corresponding SEM images of strained Ge nanowires are presented in Figure 9b. Arman Ayan et al. used COMSOL software to develop a new approach based on electrostatic actuation, gaining an axial strain of over 4%; the scheme is presented in Figure 9c,d [80]. When different voltages are applied between the Si substrate and Ge, positive and negative charges attract each other, establishing an electrical force that deflects the nanobeam.

Table 2 provides a comparison of lasing performance for devices fabricated by various strain methods. Mechanical manipulation and geometric amplification can achieve the highest strains. Currently, the most promising scheme for monolithically integrated Ge lasers relies on geometric amplification, whereas other strain methods focus on strain limits and fundamental studies rather than lasing performance.

## 4. Strained Ge Nanowire Lasers and Light-Emitting Diodes

Tensile strain modifies Ge from an indirect to a direct bandgap, enabling the feasibility to realize efficient on-chip lasers, thereby facilitating the development of monolithic photonic integrated circuits (PICs). In 2006, Rune S. Jacobsen et al. demonstrated that strained silicon could serve as an effective electro-optic material, thereby attracting increasing attention from researchers in this regime [75,76,81,82]. In the early period (mostly before 2011), the tensile strain applied to Ge films was limited, although numerous related papers have been published [83,84,85,86,87]. The intensive studies of Ge nanowire lasers under high tensile strain began in the last decade. Table 3 summarizes the brief development history of strained Ge light-emitting sources and aligns with the common development track of a novel technology. At an early stage, scientists fabricated bare, strained nanowires and characterized the enhancement of light emission via optical pumping. Afterward, they integrated the resonant cavities into the high-performance optical pumping lasers and developed electrically driven light-emitting sources to replace optical pumping. In the following contents, we will introduce and analyze four typical cases related to the resonant reflector mirrors, PL, and EL.

**Figure 10:**
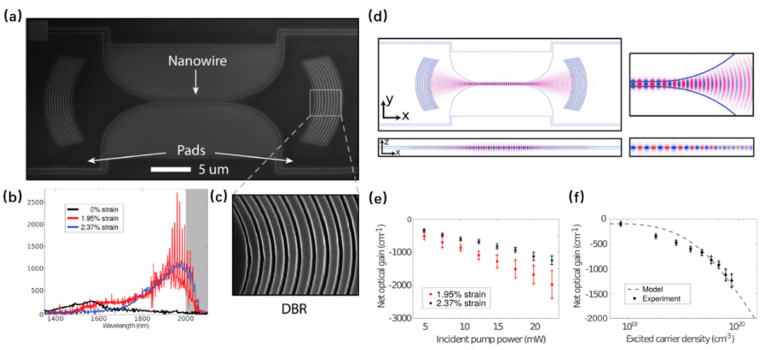
(**a**) SEM image of the suspended Ge micro-structure with the optical resonance cavity and (**c**) the corresponding detail view of the resonances. (**b**) Photoluminescence (PL) spectra of the strained Ge nanowires under various tensile strains. (**d**) Electronic field distribution of light simulated from the FDTD method. (**e**) Net optical gain of strained nanowires as a function of excited power. The black and red points represent 2.37% and 1.95% strained nanowires, respectively. (**f**) Net optical gain of the 2.37% strained Ge nanowires as a function of injected carrier density. All Panels adapted with permission from Ref. [69]. Copyright © 2016, American Chemical Society.

In these configurations, the lasing mechanisms include the population inversion within the strained Ge gain medium via external pumping and the optical feedback of the resonant cavity. The Ge gain medium offers the possibility of amplification, whereas the resonant cavity controls the lasing threshold and the specific modes. The parameter Q factor (i.e., quality factor) normally describes the ability of the resonating cavities; that is, a higher Q factor corresponds to a lower rate of energy loss and longer oscillation time. In this view, integrating high-Q resonant cavities is a key step toward realizing a high-performance on-chip light source. In 2016, Donguk Nam et al. coupled adjacent distributed Bragg-reflector (DBR) mirrors to the strained Ge laser, as shown in Figure 10a,c [69]. The Q-factor of the optical cavities was estimated to be greater than 10^4^ using finite-difference time-domain (FDTD) optical simulations. Figure 10b demonstrates photoluminescence (PL) spectra of strained Ge nanowires under various tensile strains. The distinct redshift of the main PL peak caused by higher strains suggests the reduction in the Ge bandgap. Figure 10d depicts the simulated electric field distribution within the strained Ge nanowire cavity. The integrated reflector mirrors provide strong optical confinement (Q-factor enhancement), significantly reducing parasitic scattering. While the field appears well contained within the nanowire, the mirrors are designed with finite reflectivity to allow for output coupling, enabling the extraction of the stimulated emission through the facets.

Figure 10e,f illustrate the net optical gain as a function of the incident pump power and excited carrier density, respectively. Apparently, the net optical gain of the 2.37% strained Ge nanowire laser is higher than that of the 1.95% strained one, suggesting the importance of strengthening the applied tensile strain. Additionally, the larger incident pump power would excite a higher carrier density, leading to a lower net optical gain. This phenomenon can be attributed to the thermal issue. Thermal modeling by Petykiewicz et al. suggests that under intense excitation [69] the theoretical peak temperature of a suspended Ge nanowire could reach 1575 K (neglecting melting), whereas an equivalent on-substrate device reaches only 526 K. While the suspended wire is physically limited by the 1211 K melting point of Ge, this comparison underscores the significant thermal management challenges inherent in suspended nanophotonic structures. The ultrahigh local temperature of the suspended Ge nanowire laser may originate from the poor thermal conductivity of the strained Ge nanowire, as discussed in Section 2.4. Excessive heat may worsen the unacceptable degradation of the laser, including the quality and photon quantity [91,92,93].

**Figure 11:**
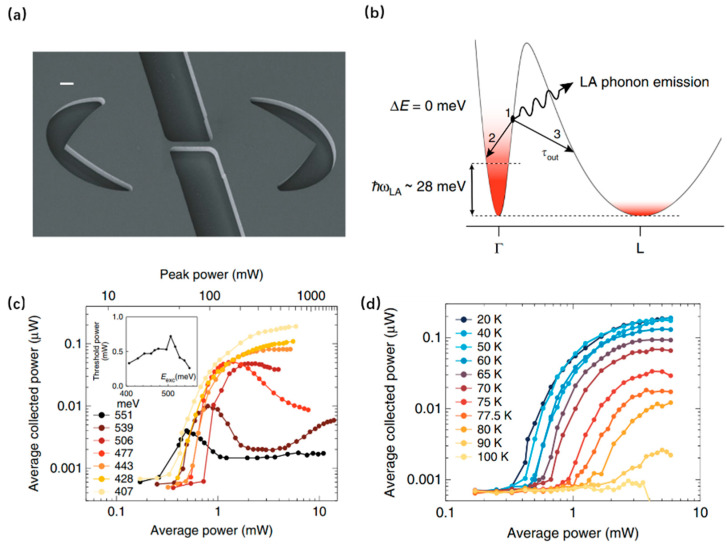
(**a**) SEM image of the suspended Ge micro-bridge nano-structure with two optical cavities on both sides. (**b**) The energy structure of the conduction band of the 5.9% strained Ge micro-bridges at low temperature. The offset between the Γ- and L- valleys is zero, which means the bottom of Γ- and L- valleys are aligned and the strain-induced direct bandgap of Ge is achieved. The numbered arrows indicate the carrier-scattering behaviors: (1) excited electrons in Γ-valleys; (2) intravalley relaxation to the Γ-valley minimum; and (3) the intervalley scattering of electrons from the Γ- to L-valley with a characteristic time τ_out_. The red shading represents the steady-state electron distribution, and ℏω_LA_ denotes the longitudinal acoustic phonon energy. (**c**) The average collected power of the 5.9% strained Ge micro-bridge as a function of the average excitation power under various excited wavelengths at 20 K. (**d**) Average collected power versus average excited power curves of the 5.9% strained Ge micro-bridge at various temperatures from 20 K to 100 K. All panels adapted with the permission from Ref. [8]. Copyright © 2019, Springer Nature. CC-BY-4.0 (https://creativecommons.org/licenses/by/4.0/).

In 2019, H. Sigg et al. constructed 280 μm-long suspended pads via patterning and etching a 〈100〉-oriented germanium-on-insulator (GOI) wafer, eventually achieving a 5.9% strained Ge micro-bridge, as depicted in Figure 11a [8]. Under such a high strain, the offset between Γ and L valley of Ge becomes zero (see Figure 11b), thereby causing the direct bandgap of 0.34 eV and the corresponding emitting peak of 3.647 µm. Figure 11c demonstrates the impact of excitation energies on the light emission of the strained Ge nanowire at a low temperature of 20 K. Different excitation energies can alter threshold pumping power and the optimized excitation energy positions by 0.407 eV. In principle, high strains could split the valence band into three bands: heavy-hole (HH), light-hole (LH), and split-off (SO) (see Scheme 1a). Thereby, it would raise the intervalence-band absorption (IVBA), also known as the free-carrier absorption (FCA) in the valence bands, which would cause a significant loss and block the development of Group-IV lasers [94,95]. Scheme 1a illustrates two possible intervalence transition paths: from the split-off band to the heavy-hole band (SO-HH) and from the split-off band to the light-hole band (SO-LH). IVBA is an unavoidable loss of the strained Ge laser. The inset of Figure 11c implies that the excitation energy of approximately 0.506 eV requires the largest pumping power, corresponding to the value of the IVBA bandgap. Figure 11d demonstrates degraded laser performance at higher temperatures and complete quenching at 100 K due to heat issues.

**Figure 12:**
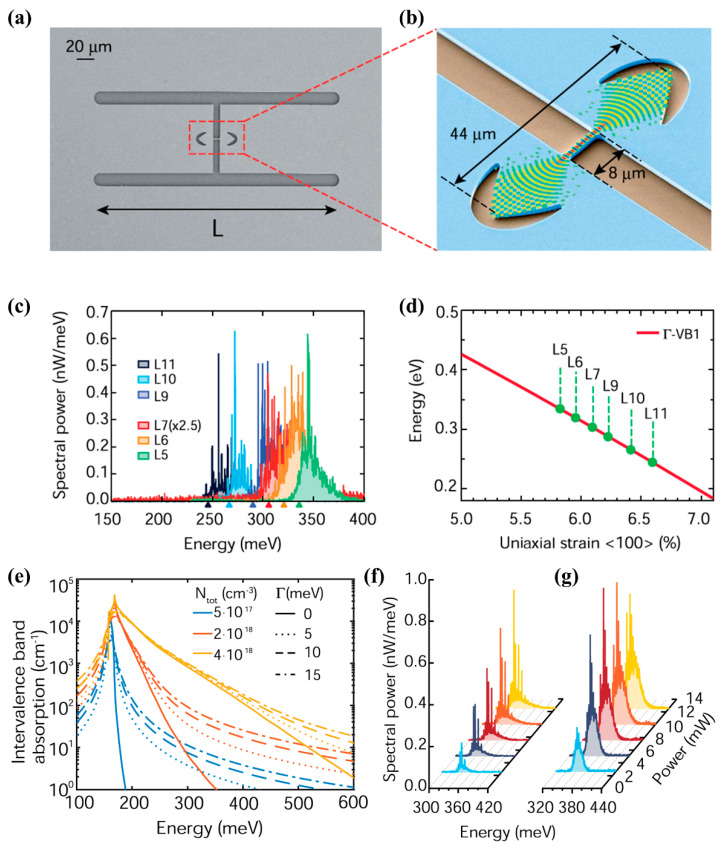
(**a**) SEM image of the on-chip Ge micro-bridge laser. (**b**) False-color SEM picture of the corner cube cavity with the corresponding simulated out-of-plane electric field distribution. (**c**) Photoluminescence spectra of various devices under different tensile strains were measured at 15 K, and the excitation power was approximately 6 mW. (**d**) The comparison between the experimental (green dots) and theoretical (the red line) Γ-bandgap at various strains. (**e**) Calculated intervalence-band absorption spectra as a function of zero-energy difference ΔE at a temperature of 30 K and different total carrier densities. (**f**–**g**) Photoluminescence spectra of phosphorus-doped Ge lasers with various doping densities of 1.8 × 10^18^ cm^−3^ (**f**) and 6.3 × 10^18^ cm^−3^ (**g**). The excitation power is set from 1 mW to 13 mW and the testing temperature is 15 K. All panels are adapted with permission from [10]. Copyright © 2022, the American Physical Society, CC-BY-4.0 (https://creativecommons.org/licenses/by/4.0/).

In 2022, F. T. Armand Pilon et al. discussed the impact of n-type doping and band offset (i.e., the energy difference between Γ- and L-bandgaps) on the performance of strained Ge micro-bridge lasers [10]. Figure 12a illustrates the free-standing Ge micro-bridge laser coupled with two corner cube reflectors. Figure 12b demonstrates the enhanced light collection efficiency enabled by the corner cube reflectors. F. T. Armand Pilon et al. fabricated different Ge micro-bridge lasers with various tensile strains (i.e., device L5-L11). In Figure 12c, the shifted peak in the photoluminescence spectra of device L5-L11 suggests the efficient bandgap adjustment enabled by tensile strain. Figure 12d shows good agreement between the theoretical prediction and experimental values for the strain–bandgap relation. Normally, intervalence-band absorption in strained Ge denotes the light absorption process between the heavy-hole and light-hole bands in the valence band. Figure 12e demonstrates a significant absorption peak at approximately 160 meV and the carrier concentration-dependent high-energy tail, due to the hole-filling effect in the reciprocal space. Figure 12f,g compare the effect of n-type doping on the lasing performance of the strained Ge micro-bridge devices. Doping can reduce material loss and enhance lasing performance at low excitation power due to a shorter lifetime caused by Auger scattering and additional gain enabled by doping-related band-tail states.

**Figure 13:**
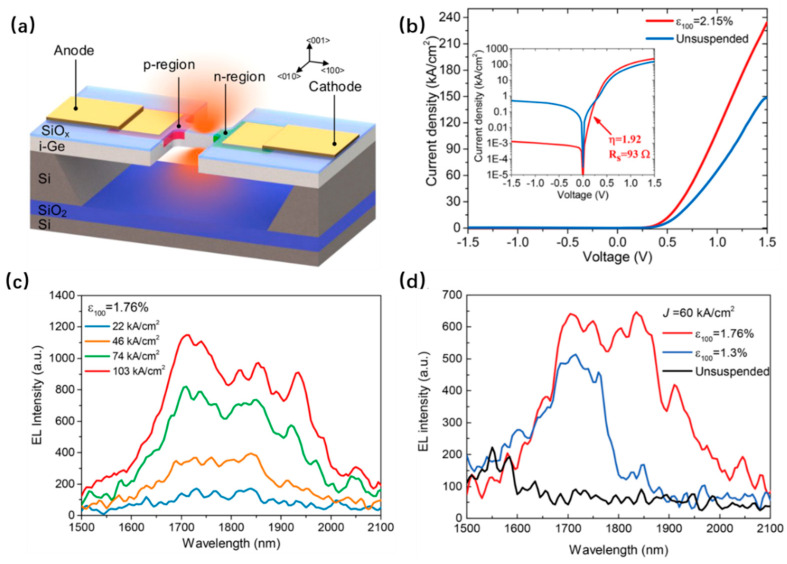
(**a**) Schematic of the suspended Ge micro-bridge light-emitting diodes (LEDs) based on a lateral p-i-n junction. (**b**) Current density (J) versus applied voltage (V) curves of the intrinsic and strained Ge LEDs. The inset is the semilog plot of the J-V characteristics. The strained Ge LEDs show better rectification behaviors. (**c**) The EL spectra of strained Ge LEDs under 1.76% driven by various current densities. (**d**) The EL spectra of the intrinsic and different strained Ge LEDs at a current density of 60 kA/cm^2^. All Panels adapted with permission from Ref. [9]. Copyright © 2019, American Chemical Society.

Junqiang Sun et al. reported an electrically driven strained Ge nanowire light source in the lateral p-i-n junction configuration, as depicted in Figure 13a [9]. The undercut Si was employed to provide a uniform uniaxial tensile stress in the suspended Ge nano-bridge. In Figure 13b, both the unsuspended and strained devices exhibit typical rectifying behavior. Compared with the unstressed device, the 1.3% strained Ge device exhibits a higher current density under positive bias and a lower series resistance, owing to doping and altered contact resistance. Figure 13c shows higher electroluminescence intensity in the strained Ge device at higher current density. Additionally, they clarified that the air/SiO_2_/Ge/air cavity could cause loss, resulting in lower quantum efficiency, as some photons could not escape the cavity. The optimal case is to realize a lasing peak with a narrow FWHM (full width at half maximum), in order to suppress parasitic spontaneous emission at non-target wavelengths and maximize the conversion efficiency into desired photons. Figure 13d depicts the emission spectrum of Ge devices under various strains. At the higher tensile strain, the bottom of the Γ valley would be lower, enabling more electron population in the Γ valley. Meanwhile, the split LH and HH valence sub-bands demonstrate larger space gaps, resulting in a broader emission peak. All these factors create higher emissions and more significant parasitic losses. Intervalence-band (IVB) absorption between LH and HH valence sub-bands depends on carrier density and can attenuate the overall gain, particularly at higher temperatures or lower photon energies.

## 5. Summary and Outlook

In conclusion, the recent advances in research on highly strained Ge nanowires/micro-bridges for on-chip laser sources have been reviewed. Although Ge is a brittle material, room-temperature plasticity may occur under high tensile strain, especially when the crystalline quality or the surfaces of Ge nanowires are not sufficiently good. Additionally, the strain–bandgap relationship becomes nonlinear at high strain, whereas the linear decrease in bandgap is observed at low strain. Similarly, the relationship between the peak shift in Raman spectroscopy and the applied strain becomes nonlinear at high strain; the same behavior can also be observed in electro-absorption spectroscopy. Furthermore, three traditional methods are described successively, including mechanical manipulation, geometric amplification, and the application of a silicon nitride stressor layer. In addition, some new approaches shown in recent years have been adopted to expand our knowledge, although some were only predicted through computer modeling and simulation. We have also discussed the advanced cases of Ge NW laser and the challenges.

Although gratifying progress has been made in these areas, realizing practical electrically pumped Ge lasers operating at room temperature remains a significant challenge. Some conventional barriers have been mentioned before, including free-carrier absorption, the optical loss caused by the too-wide emission peaks, and excessive temperature during work. The highly dense integration of strained Ge nanowire lasers into the present Si chip is another critical challenge to achieve widespread application as well. The increased tensile strain would narrow the bandgap, leading to a continuous redshift of the emission peak. At high strain magnitudes, this redshift can move the emission wavelength from the short-wave infrared (SWIR) into potentially the mid-infrared (MIR) regime, limiting the applicability of highly strained Ge lasers within the standard optical communication window (1260~1625 nm). The strain-induced direct bandgap of 〈111〉-oriented Si nanowires can remain ~0.8 eV, making them a good alternative. Nevertheless, a fundamental study of highly strained Ge nanowires is also necessary, as it can provide experience for future studies of highly strained Si nanowires.

### 5.1. Critical Comparative Analysis of Scalability

Beyond these fundamental material and optical constraints, the transition from laboratory prototypes to commercialized on-chip light sources hinges on the industrial viability and throughput of the fabrication processes. Therefore, it requires a benchmark to compare the scalability of different mass-production fabrication (Table 4). As depicted in the analysis, mechanical manipulation provides a powerful approach for fundamental research but a challenge for mass-production CMOS integration. In contrast, geometric amplification and stressor layers exhibit more promise for photonic integrated circuits. For practical applications, a “hybrid” approach combining the stressor layer with geometric amplification may be necessary to realize a compact, monolithic strained Ge laser at room temperature.

### 5.2. Challenges in Reliability and Thermal Management

A critical bottleneck for the practical application is the thermal management of suspended Ge nano-structures. For mass production, high strain typically requires undercutting the Ge micro-bridge, enabling geometric amplification. Such suspended Ge nano-structures are isolated from the substrate, severely limiting heat dissipation and leading to significant self-heating during laser operation. As discussed previously, the tiny geometric size and tensile strain can fundamentally suppress the thermal conductivity. Therefore, the combination of poor heat dissipation and high mechanical stress may worsen brittle fracture during laser processing and shorten the lifespan. In this perspective, a hybrid approach, such as strained GeSn alloying, presents a feasible strategy to lower the critical strain threshold required for a direct-bandgap transition. Therefore, it can weaken thermal-mechanical stress, thus increasing the operation lifetime and prompting the reliability of monolithic Group IV integrated lasers.

### 5.3. Alternative Promising Group IV Integrated Lasers

Aside from strained Ge on-chip lasers, some other types of Group IV integrated lasers are also promising, such as

(1) GeSn lasers. Germanium–tin (GeSn) alloys can be monolithically and epitaxially grown on the silicon substrates, suggesting good CMOS compatibility. Additionally, GeSn would become a direct-bandgap semiconductor, enabling mid-infrared light emission, once the Sn content exceeds 8%. The first GeSn laser was realized by adding 12.6% Sn content and worked at 90 K [96]. Then, the first near-room-temperature GeSn laser was fabricated with 20% Sn composition [97]. Currently, SiGeSn/GeSn heterostructure and quantum-well lasers are the main investigation direction to achieve a lower excitation threshold and higher working temperature [98].
(2) Hexagonal SiGe laser. As mentioned before, Si, Ge and SiGe in the standard cubic-diamond crystal structure should be the indirect-bandgap semiconductors, which are not suitable for the radiative recombination of carriers and further the lasing application [99,100]. The hexagonal crystal enables a direct bandgap of SiGe, corresponding to an emission peak from 1.8 μm to 2.4 μm. Nevertheless, SiGe currently requires a GaAs substrate as the growth template, limiting its compatibility with the standard CMOS process.

To place these technologies in perspective with strained Ge, Table 5 provides a benchmark matrix of their performance and compatibility.

Finally, the ultimately “holy grail” of realizing room-temperature, electrically pumped monolithic Group-IV integrated lasers may not hinge upon a single technology but on a hybrid approach. Suspended GeSn alloy nano-structures coupled with high-Q optical cavities can simultaneously lower the mechanical threshold for direct-transition emission while maintaining CMOS compatibility, holding promise for enabling 100 GHz bandwidth applications in next-generation photonic integrated circuits (PICs).

## Figures and Tables

**Table 1:** The critical strain required for the indirect-to-direct transition according to the theoretical prediction.

Year	Crystalline Orientation			Ref.
	〈100〉	〈110〉	〈111〉	
2009	7.4%	---	4.2%	[4]
2014	4.6%	---	---	[45]
2014	8.56%	Not possible	5.69%	[46]
2015	4.2%	Not possible	3.7%	[47]
2018	4.38%	Not possible	Not possible	[35]
2018	5.7%	Not possible	5.38%	[35]
2021	4.2%	Not possible	3.7%	[48]

The symbol “---” represents that no relevant information is given by the reference.

**Table 2:** Performance metrics of strained Ge lasers fabricated by various methods.

Fabrication Method	Maximum Strain	Lasing Threshold	Maximum Operating Temperature	Ref.
AFM manipulation (Mechanical manipulation)	~2.0%	---	---	[68]
3-Point bending (Mechanical manipulation)	2.5%	---	Room temperature	[5]
Micro-electro-mechanical system (Mechanical manipulation)	16% (Si)	---	Room temperature	[19]
Geometric amplification	5.7%	3.0 kW/cm^2^	Room temperature	[8]
External stressor layers	1.48%	---	Room temperature	[77]
Laser shock straining	~4.0%	---	---	[79]
Electrostatic actuation	~4.0%(Sim.)	---	---	[80]

The symbol “---” means that the relative parameter is not mentioned in the literature. “Si” indicates that the material used in the work is silicon rather than germanium. “Sim.” demonstrates a simulation rather than an experiment.

**Table 3:** The summary of strained Ge lasers.

Year	Strain	Orientation	Doping	Pumping	Temperature	Optical Cavity	Emission Peak	Optical Net Gain	IQE	Ref.
2012	0.84%	〈100〉	undoped	PL	RT	no	1860 nm	---	---	[88]
2013	3.1%	〈100〉	undoped	PL	RT	no	2066 nm	---	---	[74]
2013	2.77%	〈100〉	undoped	PL	RT	no	>2100 nm	---	---	[6]
2014	2.98%	〈100〉	undoped	PL	RT	no	2033 nm	---	---	[45]
2014	1.6%	〈100〉	undoped	PL	RT	no	1800 nm	---	---	[89]
2016	1.95%	---	n-type	PL	RT	no	~1950 nm	~−600 cm^−1^	---	[69]
2016	2.37%	---	n-type	PL	RT	BR mirrors	>2000 nm	~−400 cm^−1^	---	[69]
2017	1.6%	〈100〉	undoped	PL	83 K	BR mirrors	1530 nm	~−415 cm^−1^	---	[90]
2019	1.76%	〈100〉	p-i-n	EL	RT	no	1885 nm	---	---	[32]
2019	5.9%	〈100〉	undoped	PL	20 K	CC mirrors	3660 nm	---	80%	[31]
2022	5.8%	〈100〉	undoped	PL	15 K	CC mirrors	3875 nm	~−87 cm^−1^	---	[10]
2022	5.8%	〈100〉	n-type	PL	15 K	CC mirrors	3875 nm	~−800 cm^−1^	---	[10]

Note: RT is room temperature. BR mirrors and CC mirrors are Bragg-reflector mirrors and corner cube mirrors, respectively. The symbol “---” means that the relative parameter is not mentioned in the literature.

**Table 4:** The scalability of various fabrication methods.

Method	Production Scalability	Footprint Density	Primary Manufacturing Bottleneck
Mechanical manipulation	low	low	Requires serial, unit-by-unit manipulation (e.g., AFM)
Geometric amplification	high	moderate	Requires large stress-collector pads (100–300 μm).
Stressor layers (SiN_x_)	high	High	Strain limited by delamination and material fracture limits.

**Table 5:** Benchmark of strained Ge against competing Group IV laser technologies.

Feature	Strained Ge	GeSn Alloys	Hexagonal SiGe
Direct Bandgap origin	tensile strain (>4~5%)	Alloying (>8% Sn)	Special crystal phase (Wurtzite)
Emission wavelength	1.5–3.0 µm (redshifts with strain)	2.0–4.0 µm (depends on Sn weight)	1.8–3.5 µm (depends on Si weight)
CMOS compatibility	High (inherent)	Moderate (Sn segregation risks)	Low (currently requires GaAs nanowire templates)
Fabrication complexity	High (requires suspended structures)	Moderate (planar epitaxy)	Very high (complex crystal phase transfer)
Key challenge	Thermal management in suspended cavities	High thresholds at room temperature; material defects	Integration on Si substrates (III–IV contamination)

## Data Availability

No new data were created or analyzed in this study.
